# Utility index and vision-related quality of life in patients awaiting specialist eye care

**DOI:** 10.1371/journal.pone.0307691

**Published:** 2024-08-12

**Authors:** Aline Lutz de Araujo, Bruna Stella Zanotto, Ana Paula Beck da Silva Etges, Karen Brasil Ruschel, Taís de Campos Moreira, Felipe Cezar Cabral, Erno Harzheim, Marcelo Rodrigues Gonçalves, Roberto Nunes Umpierre, Fabiana Carvalho, Rodolfo Souza da Silva, Carisi Anne Polanczyk

**Affiliations:** 1 Department of Ophthalmology and Visual Sciences ‐ Escola Paulista de Medicina, Universidade Federal de São Paulo (UNIFESP), São Paulo, Brazil; 2 Instituto de Avaliação de Tecnologias em Saúde ‐ Universidade Federal do Rio Grande do Sul (UFRGS), Porto Alegre, Rio Grande do Sul, Brazil; 3 Post Graduate Studies Program in Epidemiology, School of Medicine ‐ Universidade Federal do Rio Grande do Sul (UFRGS), Porto Alegre, Rio Grande do Sul, Brazil; 4 Escola Politécnica ‐ Pontifícia Universidade Católica do Rio Grande do Sul, Porto Alegre, Rio Grande do Sul, Brazil; 5 Hospital Moinhos de Vento ‐ Porto Alegre, Porto Alegre, Rio Grande do Sul, Brazil; 6 Núcleo de Telessaúde do Rio Grande do Sul (TelessaúdeRS-UFRGS) ‐ Universidade Federal do Rio Grande do Sul (UFRGS), Porto Alegre, Rio Grande do Sul, Brazil; Federal University of Sao Carlos: Universidade Federal de Sao Carlos, BRAZIL

## Abstract

**Objectives:**

This study aimed to ascertain utility and vision-related quality of life in patients awaiting access to specialist eye care. A secondary aim was to evaluate the association of utility indices with demographic profile and waiting time.

**Methods:**

Consecutive patients that had been waiting for ophthalmology care answered the 25-item National Eye Institute Visual Function Questionnaire (NEI VFQ-25). The questionnaire was administered when patients arrived at the clinics for their first visit. We derived a utility index (VFQ-UI) from the patients’ responses, then calculated the correlation between this index and waiting time and compared utility across demographic subgroups stratified by age, sex, and care setting.

**Results:**

536 individuals participated in the study (mean age 52.9±16.6 years; 370 women, 69% women). The median utility index was 0.85 (interquartile range [IQR] 0.70–0.92; minimum 0.40, maximum 0.97). The mean VFQ-25 score was 70.88±14.59. Utility correlated weakly and nonsignificantly with waiting time (-0.05, *P* = 0.24). It did not vary across age groups (*P* = 0.85) or care settings (*P* = 0.77). Utility was significantly lower for women (0.84, IQR 0.70–0.92) than men (0.87, IQR 0.73–0.93, *P* = 0.03), but the magnitude of this difference was small (Cohen’s d = 0.13).

**Conclusion:**

Patients awaiting access to ophthalmology care had a utility index of 0.85 on a scale of 0 to 1. This measurement was not previously reported in the literature. Utility measures can provide insight into patients’ perspectives and support economic health analyses and inform health policies.

## Introduction

Access to services and the time it takes people to obtain treatment are relevant indicators of the health system quality and efficiency [1–3]. Brazil’s public healthcare system, the Sistema Único de Saúde (SUS), ensures health coverage for the entire population of more than 210 million people. The SUS is predicated on the principles of universality, equity, and comprehensiveness. Despite its inclusive design, the system encounters significant challenges in providing adequate specialty care. The system is based on Primary Care with a well-established family medicine model that provides preventative care and visits for general health concerns. If patients need care that is out of primary care scope, they are referred to specialty care [4, 5]. Nevertheless, the system’s specialty care does not supply enough services to cover the demand. The inadequacy in service provision has resulted in long waiting times and considerable delays in diagnosis and treatments. The time lapse between a primary care referral and an initial consultation at a specialized center can span from several months to years, compromising patient outcomes and system efficacy. Previous research has highlighted restricted availability of ophthalmology services. In two Brazilian cities—one rural and one a state capital—17.5% and 18.9% of individuals requiring eye care were unable to access it, respectively [6, 7].

This situation often results in patients experiencing psychological distress and clinical deterioration before receiving care [3]. Conversely, individuals with private health insurance have direct and prompt access to specialty services, which exemplifies the disparities within the Brazilian healthcare landscape [8, 9]. Such inequities and restricted access to healthcare services necessitate the formulation and implementation of targeted public health policies, alongside refined frameworks for priority setting and resource allocation [10].

Resource allocation strategies are increasingly being determined by how individuals assign value to health and their preferences for different health states [11, 12]. Unlike measures that determine health level based on clinical parameters, preference-based discrimination incorporates the values people ascribe to each of a series of outcomes [12]. Utility, or the utility index, is a measure associated with quality of life-based on people’s preference for living in a given state of health [12]. Utility is often used in economic health analyses, such as cost-effectiveness studies, through the quality-adjusted life years (QALY) metric [13]. To assess utility in ophthalmology studies, the VFQ-UI (Visual Function Questionnaire-Utility Index) can be used. The VFQ-UI, derived from the NEI VFQ-25 questionnaire, assesses vision-related quality of life, quantifying the impact of visual function on daily activities and overall well-being. It includes items on general vision, near and distance activities, and ocular pain, providing a comprehensive evaluation of how visual impairments affect individuals’ lives [14].

Prior studies have evaluated the utility in particular eye conditions [15–18]. Yet, a comprehensive literature review yielded no publications investigating this parameter among patients awaiting eye care, that is, before accessing diagnosis and treatment.

### Aims of the study

This study aimed to assess the utility in individuals referred by primary care to a specialized ophthalmology service. Additionally, as a secondary objective, we aimed to investigate the relationship between waiting times, utility index, and demographic predictors influencing user perception.

## Methods

### Study design

We conducted a health utility study. This study report follows the CREATE guideline for reporting utility assessments [19]. The checklist is available in S1 File.

### Population and settings

Four public health services were selected for recruitment of participants: two in the state capital of Rio Grande do Sul (Hospital de Clínicas de Porto Alegre and Hospital da Restinga e Extremo Sul, both in Porto Alegre), the southernmost state of Brazil, and two in smaller inland municipalities (Outpatient Specialty Clinic and Municipal Health Foundation, both in Santa Rosa, Rio Grande do Sul). Patients waiting for a first ophthalmologist visit were recruited from these facilities. These patients were referred due to ocular complaints. The inclusion criteria were: be a first-visit patient at an adult ophthalmology outpatient clinic and consent to participate in the study (which included agreeing to answer a 25-item quality of life questionnaire). The exclusion criteria were cognitive difficulty preventing comprehension of the study instrument and already being under the care of an ophthalmologist. Data collection took place from December 2019 to January 2020; during this period, all patients who met the criteria were invited to participate.

### Data collection

We applied the interviewer-administered version of the 25-item National Eye Institute Visual Function Questionnaire (NEI VFQ-25) questionnaire previously translated and validated for use in Brazil [20, 21]. The questionnaire consists of 25 items comprising 12 subscales, 11 of which are vision-related (global vision, difficulty with near-vision activities, difficulty with distance-vision activities, limitations in social functioning due to vision, role limitations due to vision, dependency on others due to vision, mental health symptoms due to vision, driving difficulties, limitations with peripheral vision, limitations with color vision, and ocular pain), as well as a general health construct. Each subscale consists of a minimum of one and a maximum of four questions. The scoring algorithm calculates results on a scale of 0 to 100, with higher scores representing better visual functioning [20].

An electronic version of the questionnaire was generated in the SurveyToGo application (Dooblo, Israel) and administered by interviewers with tablet computers. Demographic data (age, sex, place of residence) and waiting time from referral to the day of the scheduled visit were also collected. External investigators collected all data before patients entered the ophthalmologist’s office.

### Statistical analysis

The data were exported from the SurveyToGo application in comma-separated values (.csv) format. Microsoft Excel® software was used to calculate the subscale scores of the NEI VFQ-25 and VFQ Utility Index (VFQ-UI). The overall NEI VFQ-25 score was determined by calculating the arithmetic mean of the scores of the 12 subscales instead of the individual items [20].

The VFQ-UI consists of a 6-item subset of the NEI VFQ-25. These items were selected for the VFQ-UI based on previous clinical and psychometric analyses [14, 22, 23]. By analyzing the content of each subscale, a health preference weighting study determined the final algorithm for scoring the VFQ-UI based on a set of hypothetical health states [14, 22]. All the NEI VFQ-25 items were recorded so that they were no longer reverse-scored.

In the Google Colab environment, we performed statistical analysis in Python using the NumPy, pandas, SciPy, Pingouin, and Matplotlib libraries. Parametric variables were described as means and standard deviations, and nonparametric ones, as medians and interquartile ranges (P75–P25). To compare nonparametric variables in independent samples, we used the Mann-Whitney *U* and Kruskal–Wallis tests. To test for correlation between nonparametric variables, we used Spearman’s rank coefficients. P<0.05 was considered significant. To represent the magnitude of the differences between groups, Cohen’s d statistic was used [24–26]. Cohen’s d expresses the difference in number of standard deviations between two measures and is calculated as:

$$d=\frac{\bar{x_{1}}-\bar{x_{2}}}{\sqrt{\frac{\left(n_{1}-1\right)s_{1}^{2}+\left(n_{2}-1\right)s_{2}^{2}}{n_{1}+n_{2}}}}$$

where: x1 and x2 are the group means of the utility score; s1 and s2 are the sample variances of the groups; and n1 and n2 are the sample sizes of the groups. Cohen’s effect size classification was used to interpret the results: values equal to or greater than 0.8 represent a large effect size; 0.8 to 0.2, a moderate effect size; and less than 0.2, a small effect size [26].

The datasets from this study were made available at: Lutz de Araujo, Aline et al. (2024). Utility Index and Vision-Related Quality of Life in Patients Awaiting Specialist Eye Care [Dataset]. Dryad. https://doi.org/10.5061/dryad.h44j0zpv3.

### Ethical approval and consent to participate

The Institutional Review Board (IRB) of Hospital das Clínicas de Porto Alegre provided ethical oversight. It approved the study protocol in accordance with the local regulatory framework and the Helsinki Declaration. Relevant approvals are under CAAE 64499316.1.0000.5327 with report number 3.724.069. All participants provided written informed consent for participating in the study.

## Results

### Participants

A total of 536 individuals participated in the study. Of these, 370 (69%) were female. The mean age was 52.9±16.6 years. Seven patients could not report how long they had been on the waiting list; these were excluded from analyses that involved the time variable (number of participants after exclusions = 529). Demographic data are presented in Table 1.

**Table 1:** Demographic data for the 536 participants.

Variable	n (%)
Age*	52.9±16.6 years
Sex	
Female	370 (69%)
Male	166 (31%)
Site	
State capital	302 (56.3%)
Other municipality	234 (43.7%)
Waiting time**	7.86 (2.0–9.0) months

* Age expressed as mean ± standard deviation.

** Time expressed as median and interquartile range for 529 patients.

### Vision-related quality of life scores

Scores for each subscale, the frequency of valid responses, and the overall NEI VFQ-25 score are shown in Table 2. Due to the low frequency of valid responses in the *“driving difficulties”* subscale, we added a modified composite score that excludes this subscale [27, 28].

**Table 2:** Overall and subscale NEI VFQ-25 scores in the studied population (n = 536).

Subscale	Valid responses	Score
General health	536 (100%)	43.00 ± 24.50
Global vision	536 (100%)	60.11 ± 18.08
Ocular pain	536 (100%)	66.32 ± 27.24
Near vision	536 (100%)	66.10 ± 25.44
Distance vision	534 (99.6%)	69.81 ± 26.18
Limitations in social functioning	535 (99.8%)	83.86 ± 22.08
Vision-related mental health	536 (100%)	63.00 ± 26.49
Role limitations	536 (100%)	66.60 ± 31.33
Dependence	536 (100%)	77.58 ± 29.98
Driving difficulties	175 (32.6%)	42.94 ± 21.90
Color vision	531 (99.1%)	89.50 ± 22.34
Peripheral vision	534 (99.6%)	75.28 ± 27.90
Overall	536 (100%)	70.88 ± 14.59
Modified overall*	536 (100%)	71.79 ± 14.94

* Excludes the “Driving difficulties” domain.

### Utility indices

**Fig 1:**
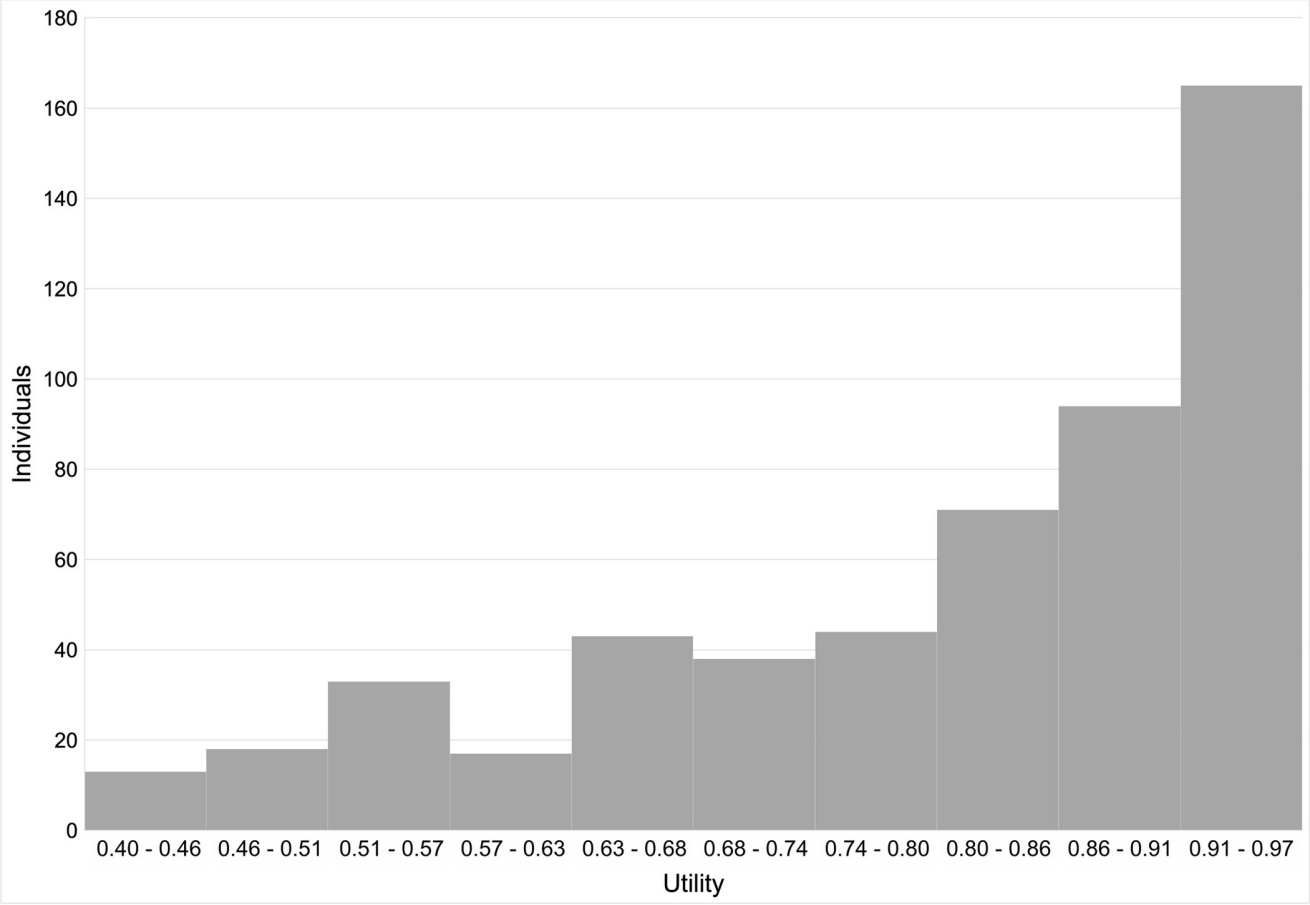
Distribution of utility values in the studied population. The x-axis shows Visual Function Questionnaire ‐ Utility Index (VFQ-UI) scores; the y-axis shows the absolute number of individuals (n = 536).

The VFQ-UI could be derived from the NEI VFQ-25 responses of all participants. The median utility was 0.85 (interquartile range 0.70–0.92), with a minimum of 0.40 and a maximum of 0.97. Fig 1 shows the distribution of utility in the study population, while Table 3 shows utility indices according to demographic characteristics.

**Table 3:** Utility indices of groups stratified by demographic characteristics, setting of care, and waiting time for specialist ophthalmology care.

	n (%)	Utility score	Cohen’s *d* effect size	P
		Median (IQR)		
Age				0.85^*^
Young adult (< 40 years)	117 (22%)	0.88 (0.77–0.91)	Reference	
Adult (40–64 years)	269 (50%)	0.84 (0.71–0.92)	0.23	
Older adult (≥ 65 years)	150 (28%)	0.81 (0.66–0.94)	0.33	
Sex				0.03^**^
Female	370 (69%)	0.84 (0.70–0.92)	Reference	
Male	166 (31%)	0.87 (0.73–0.93)	0.13	
Setting of care				0.77^*^
State capital	302 (56%)	0.85 (0.71–0.92)	Reference	
Other municipality	234 (44%)	0.84 (0.68–0.92)	0.09	
Waiting time				0.48^**^
< 6 months	190 (36%)	0.86 (0.72–0.93)	Reference	
6–11 months	236 (45%)	0.85 (0.73–0.92)	0.00	
12–23 months	43 (8%)	0.82 (0.66–0.92)	0.14	
24 months	60 (11%)	0.80 (0.60–0.92)	0.26	

^*^: Kruskal–Wallis test

^**^: Mann–Whitney *U* test, two-tailed.

IQR, indicates interquartile range.

To assess whether utility was affected by time spent on the waiting list, we calculated the correlation between utility index and waiting time. Spearman’s correlation coefficient was negative and near-zero (rho = -0.05, *P* = 0.24), indicating an inverse correlation of minimal magnitude and nonsignificant. The correlation between waiting time and each subscale score was also near-zero and devoid of statistical significance.

## Discussion

In this study, we utilized a utility index derived from the NEI VFQ-25 to determine utility in a population awaiting ophthalmological care. The median utility was 0.85 on a scale of 0 to 1. This index differed statistically between men and women, being lower in women. The median waiting time from referral to the day of the visit exceeded seven months. Patients’ utility index did not correlate with waiting time.

Patients who are waitlisted for specialty care have their utility indices affected by their health condition (e.g., visual capacity impaired by cataracts) as well as by gradual impairment and dissatisfaction associated with waiting itself, known as disutility [29]. Disutility expresses the decrease in utility that occurs due to a particular state. It is a negative value representing the impact of the given state or situation (in this case, being on a waiting list for a medical visit) on the individual’s utility [29].

Previous studies have assessed the level of disutility associated with waiting time. Waitlisting is believed to cause emotional symptoms, such as anxiety and fatigue, which have a negative impact on utility [30, 31]. On the other hand, it has been argued that waiting can have positive effects on utility due to feelings of hope concerning the expected treatment [31]. Additionally, the utility or disutility associated with waiting differs according to the type of treatment. Waiting time for orthopedic surgeries of the knee and hip, for instance, has been shown to have negative effects on patient-reported outcomes, while waiting for the treatment of varicose veins and inguinal hernia has not [2].

Measures of utility for eye diseases have been described in the literature [15–18, 23, 32]. However, there are no previous publications on the utility indices of patients on a waiting list for their first eye care appointment. Studies of patients waiting for cataract surgery have reported utility and other related aspects, such as quality of life, willingness to pay, and maximum acceptable waiting time [33–37]. The average utility assessed by the EuroQol five-dimensional questionnaire (EQ-5D) in patients awaiting cataract surgery for 16.4 ± 10.9 weeks was 0.8 ± 0.2 (median, 0.8) [35]. Willingness to pay is an alternative measure to QALY, used in cost-benefit studies [13]. Patients have reported a willingness to pay to reduce their waiting time for cataract surgery in several countries [33]. However, this measure is systematically related to individuals’ economic resources [13]; therefore, its generalizability across different contexts is limited.

When comparing utility rates between demographic groups, we found a significant difference between men and women, with lower values in women. Evidence shows that women tend to pay more attention to eye symptoms and follow medical prescriptions and advice more carefully [38]. However, our analysis of effect size showed only a minimal effect–less than 0.2, according to Cohen’s classification [26]; thus, the difference magnitude is probably devoid of clinical relevance.

The NEI VFQ-25 is the most widely used instrument to assess the vision-related quality of life [20]. The VFQ-UI is derived from a selection of NEI VFQ-25 items and aims to reflect health states based on the preferences of individuals [14]. We used the VFQ-UI to determine utility in the population of interest. The VFQ-UI has the advantage of being derived from the scores of an instrument widely used in clinical research, the NEI VFQ-25. We selected the VFQ-UI for this study due to its ability to reflect patients’ perspectives while considering visual function. Conversely, previous studies have found that the EQ-5D lacks sensitivity to discriminate vision-related activities [32, 39]. For example, substantial vision loss did not affect EQ-5D scores in patients with age-related macular degeneration [32].

One limitation of the present study is that waiting time was estimated by the participants and not objectively retrieved from the referral system. Therefore, we used an estimate that may have been affected by the participants’ recall. Another possible limitation was not having access to the patient EHR after their ophthalmology visit. If we had, we could have characterized the population in more details, including the level of visual impairment, disease type, among others.

Further research on utility across disease types can be valuable. Future studies could explore how utility during waiting time varies across different ophthalmic conditions, such as glaucoma, diabetic retinopathy, and age-related macular degeneration, each presenting unique challenges to patients’ quality of life and treatment outcomes. Additionally, investigating the impact of interventions, such as timely access to specialist care or alternative care pathways, on patient utility could provide insights into optimizing healthcare delivery for ophthalmological conditions. Understanding these factors could enhance our ability to prioritize and allocate resources effectively, ultimately improving patient outcomes and satisfaction in ophthalmic care settings.

Resource allocation and prioritization processes must be transparent and consider the perspectives of patients, physicians, payors, and the public. The present study contributes to the decision-making process by providing the vision-related quality of life and utility index in patients awaiting eye care in Brazil. Such measurements are relevant for health economics analyses and can provide insight into patients’ perspectives and preferences.

## Supporting information

S1 File CREATE checklist. (DOCX)
