# Supplementary material for: Utility index and vision-related quality of life in patients awaiting specialist eye care
Source: PLoS One. 2024 Aug 12;19(8):e0307691. doi: 10.1371/journal.pone.0307691 (PMC11318885; doi:10.1371/journal.pone.0307691)
Supplement: S1 File — (DOCX) [file pone.0307691.s001.docx]

**S1 File.** CREATE checklist.

| **Item no.** | **Section/item** | **Yes** | **No** |
| --- | --- | --- | --- |
| Descriptive system | | | |
| 1 | The attributes of the instrument are described | □ | □ |
| 2 | The number of levels in each attribute of the instrument is described | □ | □ |
| Health states valued | | | |
| 3 | The approach to selecting health states to be valued directly is explained | □ | □ |
| 4 | The number of health states valued per respondent is stated | □ | □ |
| 5 | Method(s) of assigning the health states to respondents are stated | □ | □ |
| Sampling | | | |
| 6 | Sample size/power calculations are stated and rationalized | □ | □ |
| 7 | Target population is described | □ | □ |
| 8 | Sampling method is stated and rationalized | □ | □ |
| 9 | Recruitment strategies are described | □ | □ |
| 10 | Response rate is reported | □ | □ |
| Preference data collection | | | |
| 11 | Mode of data collection is stated | □ | □ |
| 12 | Preference elicitation technique(s) are described | □ | □ |
| Study sample | | | |
| 13 | Reasons for excluding any respondents or observations are provided | □ | □ |
| 14 | Characteristics of respondents included in the analysis are described | □ | □ |
| Modeling | | | |
| 15 | The dependent variable for each model is stated | □ | □ |
| 16 | Independent variables for each model are explained | □ | □ |
| 17 | Model specifications are provided | □ | □ |
| 18 | Model estimators are described | □ | □ |
| 19 | Goodness-of-fit statistics for each model are reported | □ | □ |
| Scoring algorithm | | | |
| 20 | Criteria for selecting the preferred model are stated | □ | □ |
| 21 | The scoring algorithm is presented | □ | □ |

*CREATE* Checklist for REporting VAluaTion StudiEs
